# Nonmicrobial Activation of TLRs Controls Intestinal Growth, Wound Repair, and Radioprotection

**DOI:** 10.3389/fimmu.2020.617510

**Published:** 2021-01-21

**Authors:** William F. Stenson, Matthew A. Ciorba

**Affiliations:** Division of Gastroenterology, Washington University School of Medicine, St Louis, MO, United States

**Keywords:** hyaluronic acid, TLR4, PGE_2_, epidermal growth Factor receptor (EGFR), LGR5+ epithelial stem cell, intestinal growth, wound repair, radioprotection

## Abstract

TLRs, key components of the innate immune system, recognize microbial molecules. However, TLRs also recognize some nonmicrobial molecules. In particular, TLR2 and TLR4 recognize hyaluronic acid, a glycosaminoglycan in the extracellular matrix. In neonatal mice endogenous hyaluronic acid binding to TLR4 drives normal intestinal growth. Hyaluronic acid binding to TLR4 in pericryptal macrophages results in cyclooxygenase2- dependent PGE_2_ production, which transactivates EGFR in LGR5+ crypt epithelial stem cells leading to increased proliferation. The expanded population of LGR5+ stem cells leads to crypt fission and lengthening of the intestine and colon. Blocking this pathway at any point (TLR4 activation, PGE_2_ production, EGFR transactivation) results in diminished intestinal and colonic growth. A similar pathway leads to epithelial proliferation in wound repair. The repair phase of dextran sodium sulfate colitis is marked by increased epithelial proliferation. In this model, TLR2 and TLR4 in pericryptal macrophages are activated by microbial products or by host hyaluronic acid, resulting in production of CXCL12, a chemokine. CXCL12 induces the migration of cyclooxygenase2-expressing mesenchymal stem cells from the lamina propria of the upper colonic crypts to a site adjacent to LGR5+ epithelial stem cells. PGE_2_ released by these mesenchymal stem cells transactivates EGFR in LGR5+ epithelial stem cells leading to increased proliferation. Several TLR2 and TLR4 agonists, including hyaluronic acid, are radioprotective in the intestine through the inhibition of radiation-induced apoptosis in LGR5+ epithelial stem cells. Administration of exogenous TLR2 or TLR4 agonists activates TLR2/TLR4 on pericryptal macrophages inducing CXCL12 production with migration of cyclooxygenase2-expressing mesenchymal stem cells from the lamina propria of the villi to a site adjacent to LGR5+ epithelial stem cells. PGE_2_ produced by these mesenchymal stem cells, blocks radiation-induced apoptosis in LGR5+ epithelial stem cells by an EGFR mediated pathway.

## Introduction

Toll family receptors were initially described as regulating development in Drosophila (1). Toll protein promotes dorsal-ventral polarity. The Toll pathway also mediates innate immunity in Drosophila. The interleukin-1-receptor (IL-1R) was the first identified mammalian homolog for Toll (2). Both Toll and IL-1R are transmembrane spanning receptors that signal through NF-κB. A directed search for other mammalian Toll homologs revealed a series of TLRs which play key roles in the innate immunity (3). TLRs bind specific structurally conserved microbial molecules termed pathogen associated molecular patterns (PAMPs), although they are produced by commensal organisms as well as pathogens. Among the PAMPs are lipoteichoic acid (LTA), a component of gram positive bacteria that binds TLR2, and LPS, a component of gram negative bacteria that binds TLR4 (4). Although the Toll family receptors are developmental proteins in Drosophila there has been no suggestion that TLRs are involved in mammalian growth and development (3). TLRs respond to PAMPs which are components of microbial agents; however, in stress states TLRs also respond to host molecules. Matzinger proposed a “danger model” in which TLRs respond to host molecules that are released or exposed during tissue injury (5). These TLR activating host molecules are characterized as “danger associated molecular patterns” or DAMPs. For example, ischemic reperfusion injury, which is common in solid organ transplantation, exposes host TLR agonists including heat shock proteins 60 and 70, high mobility group box 1(HMGP1) and hyaluronic acid (HA) (6–8). TLR activation by these DAMPs induces a sterile inflammatory response (8).

**Figure 1:**
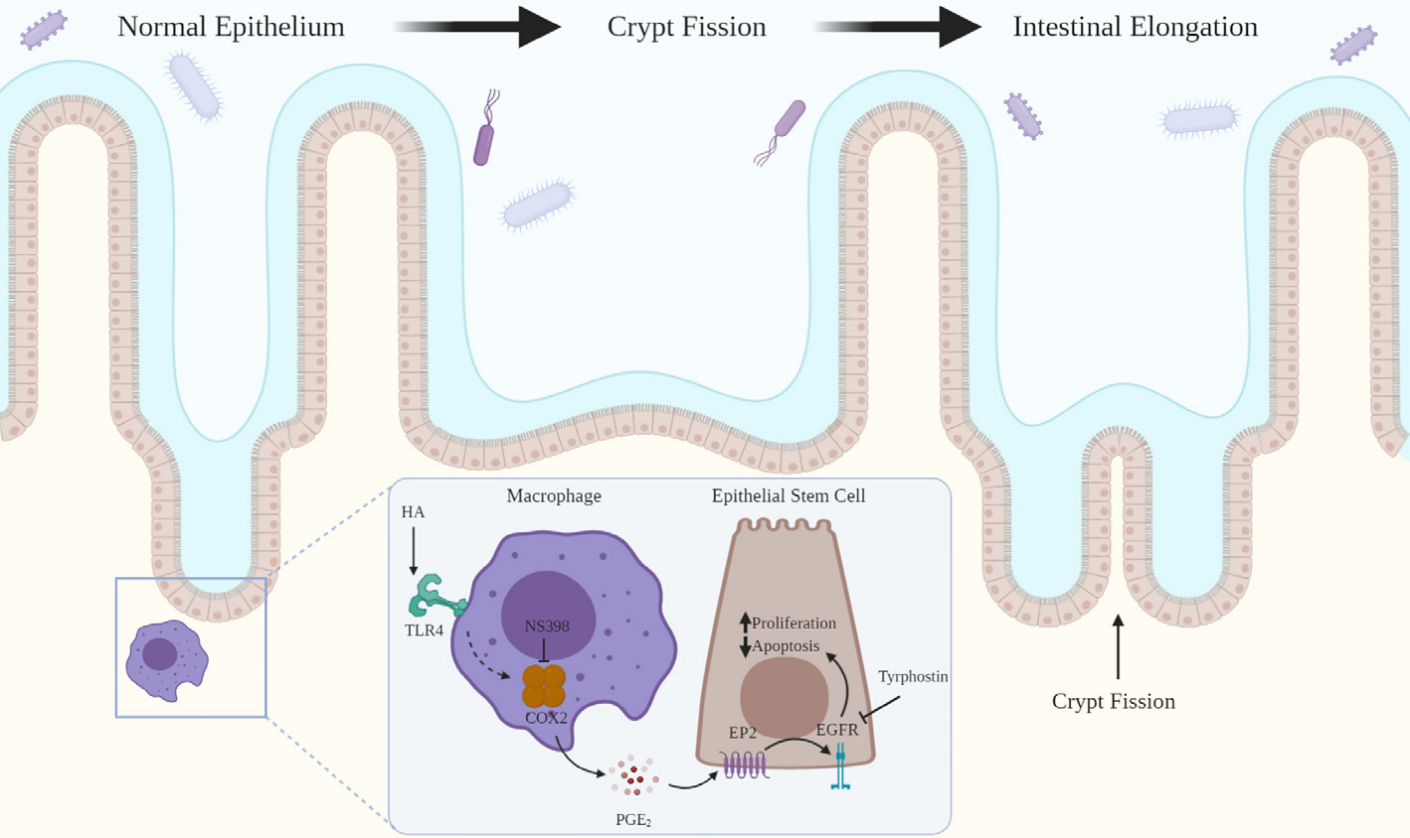
HA binding to TLR4 drives intestinal growth. HA binding to TLR4 on pericryptal macrophages results in COX-2 mediated release of PGE_2_ which binds to EP2 on LGR5+ epithelial stem cells. This transactivates EGFR promoting LGR5+ stem cell proliferation and blocking apoptosis. LGR5+ stem cell proliferation leads to crypt fission and intestinal elongation. Inhibition of COX-2 (NS-398) or EGFR (tyrphostin) decreases LGR5+ stem cell proliferation and crypt fission.

**Figure 2:**
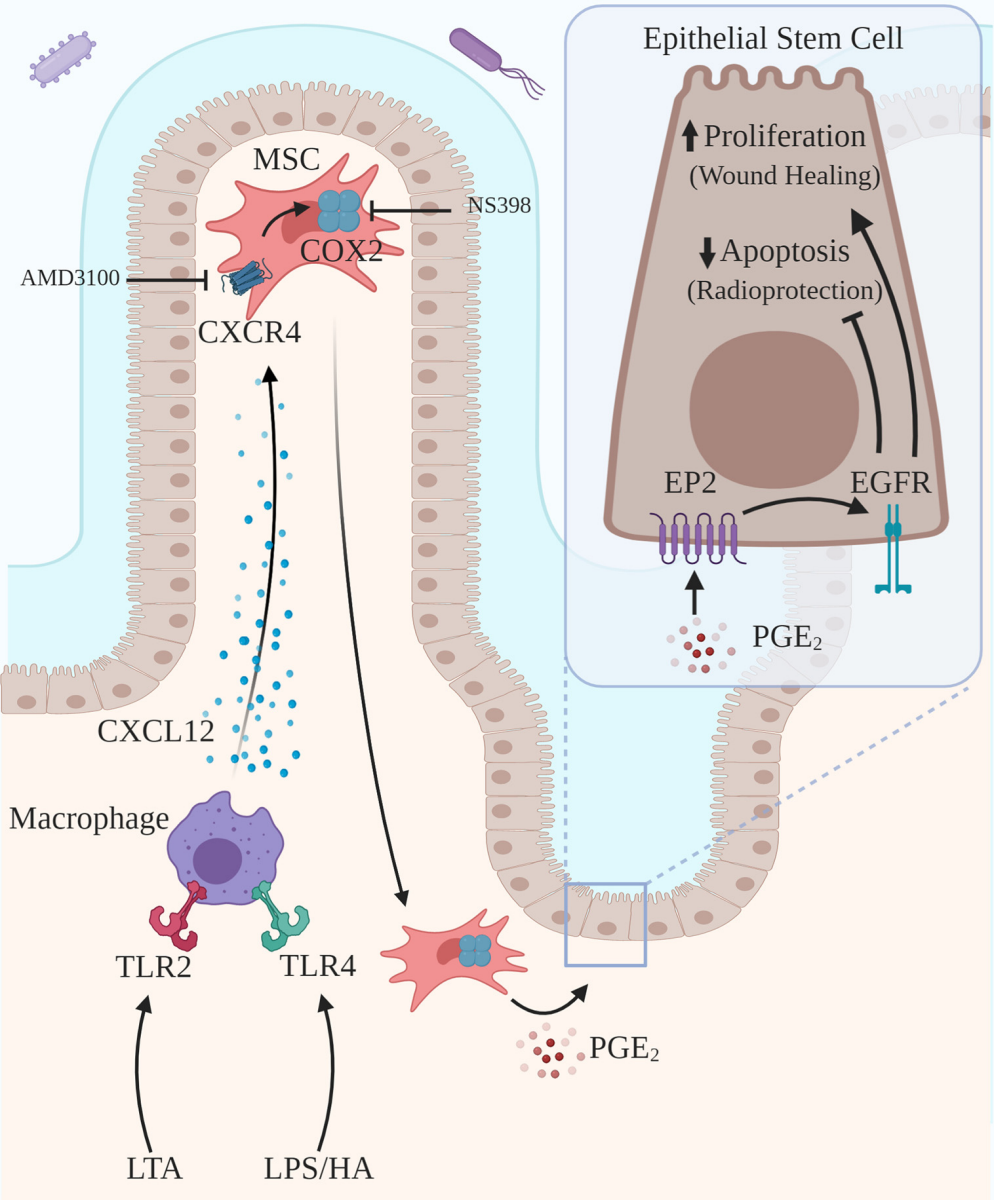
Activation of TLR2/TLR4 on pericryptal macrophages drives wound healing and radioprotection. Pericryptal macrophages express TLR2 and TLR4. TLR2/TLR4 agonists, including LTA, LPS and HA, drive epithelial proliferation as part of wound healing. Exogenous TLR2/TLR4 agonists, including LTA, LPS, and HA, induce radioprotection by blocking radiation-induced apoptosis. Activation of TLR2 by LTA or activation of TLR4 by LPS or HA results in the release of the chemokine CXCL12, which binds to CXCR4 on COX-2 expressing MSCs. Activation of CXCR4 results in the migration of the MSCs to a site adjacent to the pericryptal macrophages and also adjacent to LGR5+ epithelial stem cells. PGE_2_ released by MSCs binds to EP2 on the LGR5+ epithelial stem cells transactivating EGFR, promoting proliferation and blocking radiation-induced apoptosis. Inhibition of CXCR-4 (AMD3100) blocks radioprotection induced by TLR activation.

This review addresses two novel related intercellular pathways in which a host molecule, HA, binding to TLR2 and TLR4 drives physiologic processes in the intestine and colon. In the first pathway (**Figure 1**), intestinal and colonic growth is regulated by endogenous HA activating TLR4 on pericryptal macrophages resulting in the release of PGE₂ which promotes LGR5+ stem cell proliferation, crypt fission and intestinal elongation. In the second pathway (**Figure 2**), wound repair and intestinal radioprotection are initiated by TLR2/4 activation in pericryptal macrophages resulting in the production of the chemokine CXCL12 which, in turn, binds to CXCR4 on cyclooxygenase 2 (COX-2)-expressing mesenchymal stem cells (MSCs) in the lamina propria of intestinal villi. The MSCs migrate to an area adjacent to the intestinal crypts and release PGE₂ which, in wound repair, promotes LGR5+ stem cell proliferation and, in radioprotection, blocks radiation- induced apoptosis in LGR5+ stem cells.

## TLR4 Signaling Regulates Epithelial Proliferation in Intestinal and Colonic Growth and in Colonic Wound Repair

In neonatal life intestinal and colonic elongation occurs through crypt fission (**Figure 1**). In crypt fission LGR5+ epithelial stem cells proliferate increasing the size of the crypt (9). The crypt then divides forming two crypts (10). Neonatal mice deficient in TLR4 have markedly diminished LGR5+ stem cell proliferation and diminished crypt fission (11). As a consequence of diminished crypt fission, adult mice deficient in TLR4 have shorter intestines and colons than wild type mice (11).

TLR signaling also regulates epithelial proliferation in the repair phase of the dextran sodium sulfate

(DSS) model of ulcerative colitis (12–14). Administration of DSS in the drinking water kills epithelial cells resulting in bacterial invasion and inflammation. Withdrawal of DSS from the drinking water initiates wound repair marked by rapid epithelial proliferation. Withdrawal of DSS from the drinking water in mice deficient in MyD88, a TLR adaptor protein, or TLR2 or TLR4, results in an impaired repair response marked by diminished epithelial proliferation, increased weight loss, and increased mortality compared to wild type mice (12). Broad-spectrum antibiotics given to wild type mice prior to DSS also decrease epithelial proliferation in the repair phase (12). Administration of either LTA or LPS reverses the negative effects of broad-spectrum antibiotics. This suggests that TLR2 and TLR4 signaling driven by PAMPs from commensal bacteria promotes epithelial proliferation during wound repair in the colon.

The demonstration that TLR4 signaling regulates epithelial proliferation in growth and wound repair raises two questions. What is the cellular location of the TLR4 signaling that drives growth and wound repair? What is the relevant endogenous TLR4 agonist in growth and wound repair?

## TLR Signaling in Pericryptal Macrophages Drives Growth and Wound Repair

TLR4 is expressed in intestinal epithelial cells, stromal cells and macrophages (15). Intestinal epithelial cells express low levels of TLR4 and are relatively unresponsive to LPS (15). TLR4 is also expressed in macrophages which are a component of the epithelial stem cell niche in the intestine and colon (16). TLR4 signaling in macrophages is required for normal growth in the intestine and colon (17).Under homeostatic conditions mice with selective deletion of TLR4 in myeloid cells have the same level of diminished crypt fission and LGR5+ stem cell proliferation as do mice globally deficient in TLR4 (17). In neonatal mice depletion of macrophages with clodronate liposomes decreases crypt fission and LGR5+ stem cell proliferation to the same levels seen in mice with selective deletion of TLR4 in myeloid cells. Colony-stimulating factor (CSF)-1 is required for macrophage development and survival. Depletion of macrophages with an antibody to CSF-1 receptor results in a marked reduction in LGR5+ epithelial stem cell proliferation (18).

TLR4 signaling in myeloid cells, particularly in pericryptal macrophages, is also required for the epithelial proliferative response in the repair phase of DSS colitis. Wild type mice transplanted with bone marrow from MyD88 deficient mice have diminished epithelial proliferation after withdrawal of DSS (14). In contrast to the markedly reduced epithelial proliferative response to DSS seen in mice globally deficient in MyD88, mice expressing MyD88 only in myeloid cells have an epithelial proliferative response to DSS withdrawal similar to wild type mice (19).

Taken together these studies suggest that TLR4 activation in pericryptal macrophages drives LGR5+ stem cell proliferation and crypt fission in intestinal and colonic growth and drives epithelial proliferation in wound repair.

## HA: Fragment Size, Receptors, and Biologic Effects

HA, a glycosaminoglycan, is a polymer made up of repeating disaccharides of N-acetylglucosamine and glucuronic acid (7, 20). HA is synthesized by many cell types including fibroblasts and smooth muscle cells (20). HA is synthesized by three HA synthases (HAS1, HAS2, and HAS3) in the plasma membrane and secreted into the extracellular space (21). HA polymers, reaching up to 10,000 kDa, form a component of the extracellular matrix. In the intestine and colon HA is found in the extracellular space in a band adjacent to crypt epithelial cells and pericryptal macrophages (22).

In the face of injury and inflammation the synthesis of HA increases and the distribution of HA in the extracellular space expands. In inflammatory diseases of the GI tract such as Crohn’s disease and DSS colitis, the distribution HA expands from a narrow band around the crypt base to extend further up the crypt and eventually to fill much of the lamina propria (22). In DSS colitis increased HA deposition induces increased HA synthesis resulting in a feed-forward loop (23).

Under homeostatic conditions most of the HA in the extracellular matrix is in the high molecular weight (HMW) form (>500 kDa) (24). In injury states HMW-HA is broken down to low MW (LMW) forms by hyaluronidases released by dying cells (5). In infectious states HMW-HA may be catabolized by microbial hyaluronidases. As inflammation is cleared and the wound heals, LMW-HA is cleared and HMW-HA once again becomes the dominant form (24). In chronic inflammation LMW-HA persists.

The biologic effects of HA are mediated primarily through receptor binding (25). HA binds to CD44, TLR2, TLR4, the receptor for HA-mediated motility (RHAMM), layilin, lymphatic vessel endothelial HA receptor- 1(LYVE-1), and HA receptor for endocytosis (26). Here we will focus on CD44, TLR2 and TLR4. HA fragments of different sizes bind to different receptors and thus have different biologic effects (27). HMW-HA is associated with health and diminished inflammation (24, 28). HMW-HA has anti-inflammatory effects in lung injury (29) and collagen-induced arthritis (30). These anti-inflammatory effects are largely mediated by HMW-HA binding to CD44. CD44 activation by HMW-HA promotes cell adhesion, lymphocytic migration and gastric epithelial cell proliferation (31, 32). Crosslinking CD44 promotes the production of the anti-inflammatory cytokines IL-2, IL-10, and TGF-β (24).

LMW-HA includes fragment sizes that are primarily protective (20–75 kDA) and smaller sizes that are proinflammatory. TLR2 and TLR4 preferentially bind to LMW-HA. TLR2 and TLR4 binding to LMW-HA promotes the production of proinflammatory cytokines including TNFα, MIP, IL-1β, IL-6, and IL-12 (24, 33–35). Binding by nonmicrobial DAMPs, including LMW-HA, results in TLR4 activation in sterile environments such as joints affected with osteoarthritis (36).

Although both LMW-HA and LPS bind to TLR4, the results of TLR4 activation by LMW-HA and LPS are not identical. TLR4 activation by LPS and LMW-HA require different accessory molecules. TLR4 activation by LPS requires a TLR4-MD2 complex, LPS binding protein, and CD14 which delivers LPS to the TLR4-MD2 complex (33, 34). In contrast, TLR4 activation by LMW-HA requires a TLR4-MD2 complex but is independent of CD14 and LPS binding protein. The presence of CD44 also enhances the effects of HA binding to TLR4 although the presence of CD44 is not required for HA activation of TLR4. Lamina propria macrophages express TLR4 and MD2 but lack CD14 and thus should be responsive to HA but not to LPS (37). Incubation of cultured monocytes with LPS and LMW-HA (4-, 6-, and 8-mer) resulted in different patterns of gene induction reflecting the differences in the accessory molecules involved in TLR4 activation (34). MH-S cells, a mouse alveolar macrophage cell line, were incubated with LPS or HA (MW not specified). LPS induced CXCR-6, and defensin-β15 to a greater extent than did HA. In contrast, HA induced SOCS3, MMP3, MMP13, TGF-β, G-CSF, GM-CSF, IL-1α, and TNF-α to a greater extent than did LPS. IL-6, iNOS, and IL-1β responded similarly to LPS and HA.

Although most studies suggest that HMW-HA binds CD44 and LMW-HA binds TLR2 and TLR4. There is evidence that LMW-HA binds both CD44 and TLR4. LMW-HA (6-mer) incubated with human chondrocytes induced the release of the proinflammatory cytokines TNFα, IL-1β, and IL-6 (31). Addition of antibodies to either CD44 or TLR4 reduced cytokine production partially, whereas addition of both antibodies reduced cytokine production further (31).

It is possible that HMW-HA blocks TLR4 activation by LPS (24). In a lung inflammation model HMW-HA is anti-inflammatory and its effects are thought to be through immobilization of LPS (29). In a colon cancer model HMW-HA binding to TLR4 on cancer cells blocks TLR4 activation by LPS (38).This raises a question as to whether endogenous HA binding to TLR4 on the basolateral surface of LGR5+ crypt epithelial cells blocks TLR4 activation by LPS.

## HA Is the Relevant TLR4 Agonist in Intestinal Growth and Wound Repair

TLR4 activation by HA drives LGR5+ epithelial stem cell proliferation and crypt fission in normal growth in the intestine and colon (11, 17). The size of HA fragments in the lamina propria of the intestine and colon under homeostatic conditions has not been addressed, but studies in other systems suggest that the HMW form predominates (24). PEP-1 is a synthetic 12-mer peptide that binds to HA and prevents its binding to its receptors (39). Intraperitoneal injection of PEP-1 from 3 to 8 weeks of age results in a 30% shortening of the intestine and colon compared with untreated mice (40). The intestine and colon of PEP-1-treated mice show an atrophic appearance with shortened crypts and villi and fewer BrdU positive epithelial cells. This suggests that endogenous HA, distributed near the crypt base, drives epithelial proliferation and normal intestinal growth. Intraperitoneal injection of exogenous HA, with a broad range of MWs up to 750 kDa, from 3 to 8 weeks of age results in a hyperplastic intestine and colon with longer crypts and villi and an increase in BrdU positive epithelial cells, but does not increase elongation (40).

In neonatal mice, administration of PEP-1 from 7 to 14 days of age decreases LGR5+ stem cell proliferation and crypt fission by 30%. Neonatal mice deficient in TLR4 have decreased LGR5+ stem cell proliferation and crypt fission compared to wild type mice (11). In mice deficient in TLR4, PEP-1 does not further reduce LGR5+ stem cell proliferation or crypt fission suggesting that TLR4 activation by endogenous HA drives LGR5+ stem cell proliferation and crypt fission. HA appears to be the only TLR4 ligand involved in promoting intestinal growth. This is in contrast to wound repair where both HA and PAMPs from commensal organisms drive epithelial proliferation through TLR2/TLR4 signaling (12). Mice deficient in CD44 have reduced LGR5+ stem cell proliferation and crypt fission compared to wild type mice but the reductions are not as great as those seen in mice deficient in TLR4 (11). This suggests that endogenous HA binding to both CD44 and TLR4 promotes intestinal growth. This may be the product of endogenous HAs of different molecular weights binding separately to CD44 and TLR4 or it may be the product of HA binding to a CD44-TLR4 complex (33, 34). Treatment of neonatal mice with PEP-1 reduces crypt fission by 30% and treatment of mice with PEP-1 from 3 to 8 weeks of age decreases intestinal and colonic length by 30% suggesting that the HA/TLR4 pathway drives 30% of intestinal and colonic growth (11, 17, 40).

It is difficult to establish the role of the MW of HA in driving intestinal growth through TLR4 activation. Under homeostatic conditions most of the HA in the extracellular matrix should be in the high MW form (35). There are suggestions that TLR4 is preferentially activated by the low MW form of HA (35, 41). Despite these suggestions there is good evidence that endogenous HA activates TLR4 and promotes growth even though most of the endogenous HA is in the high MW form (11, 17, 40). Although some studies have used defined MW preparations of HA (35kDa) (42, 43), most studies of the effects of exogenous HA on growth, wound repair and radioprotection have used an HA preparation with a broad range of MWs up to 750kDa (17, 22, 40, 44). Repeating these studies with defined MW preparations of HA would give some insight into the role of MW in these biologic effects of HA.

TLR4 activation by HA also plays a role in wound repair (22). Exogenous HA, in a preparation with a broad range of MWs up to 750 kDa, is protective against DSS colitis in wild type mice but not in mice deficient in MyD88, TLR4, or COX-2 (22). In wild type mice exogenous HA is also therapeutic in established DSS colitis. The severity of DSS colitis is assessed by weight loss, disease severity score, and histologic index. Using these criteria DSS colitis is more severe in TLR4 deficient mice than in wild type mice. Coadministration of PEP-1 and DSS to wild type mice results in a worse disease severity score and a worse histology score compared to DSS alone. However PEP-1 does not further worsen the scores in mice deficient in TLR4. This suggests that TLR4 activation by endogenous HA promotes healing in DSS colitis. However, previously referenced studies demonstrated that broad spectrum antibiotics worsen DSS colitis and that these effects are rescued by LTA and LPS suggesting that PAMPs from commensal organisms also promote healing in DSS colitis (12).

Taken together these studies addressing the cellular location of the TLR4 signaling that drives growth and wound repair and the nature of the relevant TLR4 ligand suggest that HA activation of myeloid TLR4 mediates intestinal and colonic growth and wound repair.

Exogenous HA has effects on the GI tract beyond growth, wound repair and radioprotection. Low MW (35kDa) increases the expression of zonula occludens-1 (ZO-1), a tight junction protein (43). HA in human milk induces defensin-2, an antimicrobial protein (45). Oral HA is protective in a mouse model of necrotizing enterocolitis (42). Exogenous HA reduces proinflammatory signaling in Kuppfer cells and protects mice from alcoholic liver disease (46). Whether these additional effects of HA on the GI tract are mediated through TLR activation and PGE₂ has not been addressed.

Mediation of wound repair by HA activation of TLRs is not unique to the GI tract. A study of pulmonary injury induced by intratracheal bleomycin demonstrates the role of HA activation of TLR4 in sterile injury (29). Bleomycin induces oxide injury resulting in the generation of low LMW-HA. Bleomycin injury also induces the migration of neutrophils into the lungs, thickening of the interstitium and epithelial cell apoptosis. In mice deficient in TLR2 and TLR4 bleomycin induces less neutrophil migration but greater thickening of the interstitium and enhanced apoptosis and mortality compared with wild type mice. Treatment of wild type mice with PEP-1 prior to bleomycin results in enhanced bleomycin-induced apoptosis similar to that seen in the mice deficient in TLR2 and TLR4. This suggests that endogenous HA binding to TLR2 and TLR4 blocks bleomycin-induced apoptosis. In contrast to DSS colitis, where commensal organisms drive the immune response, bleomycin injury is a sterile process. Thus, it is easier to make the case that HA is the only relevant TLR2/4 agonist in bleomycin injury.

## PGE₂ Produced Downstream From TLR4 Activation Mediates Intestinal Growth and Wound Repair

In the intestine PGE₂ promotes epithelial proliferation and blocks epithelial cell apoptosis (47, 48). PGE₂ upregulates LGR5 in colon cancer (49). PGE₂ is also radioprotective in the intestine (50). PGE₂ has its biologic effects through binding four receptors: EP1, EP2, EP3, and EP4 (51). In many cases the rate limiting step in PGE₂ production is the release of arachidonic acid from cellular phospholipids by the activation of phospholipase A2 (PLA2) (52, 53). Arachidonic acid is metabolized to PGE₂ and other prostanoids through COX-1 and COX-2. COX-1 is expressed constitutively in many cells and produces prostanoids under homeostatic conditions. COX-2 expression is induced in macrophages and other cell types in response to cytokine activation during stress states. Although COX-2 expression is typically induced in stress states, in neonatal mice there is a population of pericryptal macrophages that express COX-2 constitutively (17). In adult mice there is a population of MSCs in the intestine and colon that expresses COX-2 constitutively (54). These COX-2 expressing MSCs are not seen in neonatal mice (17).

Endogenous PGE₂ produced through COX-2 mediates crypt fission and LGR5+ stem cell proliferation during neonatal growth in the intestine (11, 17). In COX-2 deficient mice, crypt fission and LGR5+ stem cell proliferation are decreased (17). Similarly, blocking endogenous PGE₂ production with NS-398, a selective COX-2 inhibitor, decreases crypt fission and LGR5+ stem cell proliferation. In neonatal mice exogenous PGE₂ promotes epithelial proliferation. Intraperitoneal administration of dimethylPGE₂ (dmPGE₂), a stable PGE₂ analog, increases crypt fission and LGR5+ stem cell proliferation to levels far above those seen at baseline. Butaprost, a selective EP2 agonist, promotes crypt fission as effectively as dmPGE₂ suggesting that these effects of PGE₂ are mediated through EP2 signaling. Thus, under homeostatic conditions, endogenous PGE₂, produced through COX2, binds to EP2 promoting crypt fission and LGR5+ stem cell proliferation. However, the levels of endogenous PGE₂ produced at baseline are not sufficient to achieve the maximal crypt fission and LGR5+ stem cell proliferation achieved with intraperitoneal dmPGE₂. PGE₂ produced through COX-2 also promotes epithelial proliferation in the repair phase of DSS colitis (22, 55). COX-2 deficient mice have diminished epithelial proliferation in the repair phase of DSS colitis. The impaired proliferative response seen in mice deficient in either TLR4 or COX-2 is rescued by intraperitoneal dmPGE₂.

## COX-2-Expressing Pericryptal Macrophages and COX-2-Expressing MSCs Are the Sites of the PGE₂ Production That Drives Intestinal Growth, Wound Repair, and Radioprotection

In neonatal mice, COX-2 expressing pericryptal macrophages produce PGE₂ that drives crypt fission and LGR5+ stem cell proliferation (**Figure 1**) (17). Macrophage depletion with clodronate in wild type neonatal mice decreases crypt fission and LGR5+ stem cell proliferation to the same degree as is seen in mice deficient in COX-2. Intraperitoneal administration of dmPGE₂ reverses the effects of both macrophage depletion and COX-2 deficiency on crypt fission and LGR5+ stem cell proliferation (17).

In contrast to neonatal mice, where growth is driven by PGE₂ produced by pericryptal macrophages, in adult mice wound repair and radioprotection are driven by PGE₂ produced by COX-2 expressing MSCs (**Figure 2**) (22, 56–58). There are COX-2 expressing MSCs in adult mice but not neonatal mice (14, 17). Under homeostatic conditions COX-2 expressing MSCs reside in the lamina propria in the villi in the intestine and in the lamina propria in the upper crypts in the colon. In response to injury or the administration of a radioprotective agent these MSCs migrate to positions adjacent to the LGR5+ crypt epithelial stem cells. The positioning of the COX-2 expressing MSCs is important in that PGE₂ has a very short half-life in tissue such that PGE₂ acts only on cells in close proximity to the PGE₂ producing cells (59, 60).

Repositioning of COX-2 expressing MSCs mediates the radioprotective effects of the probiotic lactobacillus rhamnosus GG (LGG). LGG releases LTA, a TLR2 agonist (57). Administration of LGG or LTA to mice results in the release of the chemokine CXCL12 by TLR2- expressing pericryptal macrophages (57). CXCL12 is a chemokine produced in response to TLR activation (61). CXCL12 binding to CXCR4 on COX-2 expressing MSCs causes them to migrate from the lamina propria in the villi to a site adjacent to the LGR5+ epithelial stem cells in the crypt base (57) (**Figure 2**). Both LGG induced radioprotection and the migration of the COX-2 expressing MSCs are TLR2 dependent. Release of PGE₂ from COX-2 expressing MSCs blocks radiation-induced apoptosis. Administration of AMD3100, an inhibitor of CXCR4 activation, abrogates both MSC migration and the radioprotective effects of LGG and LTA (57).

The migration of COX-2 expressing MSCs is also involved in wound repair. In response to DSS-induced injury in the colon, COX-2 expressing MSCs migrate from the lamina propria in the upper crypt to the lamina propria in the lower crypt (14). Both MSC migration and epithelial proliferation in response to DSS are MyD88 dependent. The chemokine that induces the migration of the COX-2 expressing MSCs in DSS colitis has not been identified but it is reasonable to think that it is CXCL12, the chemokine that induces the migration of COX-2 expressing MSCs in response to LGG and LTA (57). Although there is strong evidence that MSCs are the source of the PGE₂ that drives wound repair and radioprotection, colonic myofibroblasts also produce PGE₂ in response to TLR activation (62).

Although PGE₂ produced through COX-2 mediates LGR5+ stem cell proliferation in both normal growth and radioprotection/wound repair, the intercellular pathways are different. Under homeostatic conditions in neonatal mice activation of TLR4 on pericryptal macrophages by endogenous HA results in the production of PGE₂ which drives LGR5+ cell proliferation. In contrast, in adult mice TLR2/TLR4 activation on pericryptal macrophages by exogenous HA or other TLR2/TLR4 agonists results in CXCL12 production resulting in the migration of COX-2 expressing MSCs. What is the basis of the apparent differences in response to TLR activation? One possibility is that CXCL12 is produced under both circumstances but in neonatal mice there are no COX-2 expressing MSCs to migrate in response to CXCL12. A possible explanation for the differential response to HA in the two circumstances may relate to the much higher dose of exogenous HA compared to endogenous HA or differences in the MW distributions of endogenous and exogenous HA. It is also possible that the involvement of different accessory molecules in TLR4 activation results in different patterns of gene expression (33, 34). Low dose, high MW endogenous HA binding to TLR4 may preferentially promote PGE₂ production, whereas high dose low MW exogenous HA or LPS or LTA binding to TLR4 may preferentially promote CXCL12 production.

## PGE₂ Promotes Intestinal and Colonic Growth and Wound Repair Through EGFR Activation

LGR5+ stem cell proliferation is mediated through both β-catenin and EGFR activation (63–66). PGE₂ can act through both β-catenin and EGFR activation (67–70). PGE₂ binding to EP2 on epithelial cells transactivates EGFR through a src family kinase mediated mechanism (48). Transactivation of EGFR by PGE₂ promotes proliferation in colon cancer cell lines (70). In human biliary carcinoma cells *in vitro*, addition of LPS initiates a positive feedback loop of TLR4 activation, PGE₂ production through COX-2 and EGFR activation (71). Both PGE₂ mediated inhibition of radiation-induced apoptosis and PGE₂ mediated promotion of crypt fission are mediated through EP2 (11, 48). Neonatal mice deficient in intestinal epithelial cell EGFR have markedly diminished LGR5+ stem cell proliferation and crypt fission under homeostatic conditions (11). Similarly, inhibition of EGFR activation with tyrphostin diminishes LGR5+ cell proliferation and crypt fission. Moreover, dmPGE₂ fails to rescue the decreases in LGR5+ cell proliferation and crypt fission associated with epithelial cell EGFR deficiency and with tyrphostin. In addition to promoting LGR5+ cell proliferation through EGFR activation, PGE₂ blocks radiation induced apoptosis in the intestine through the same mechanism (48). Although the evidence suggests that PGE₂ promotes LGR5+ proliferation in the intestine through EGFR activation it is also possible that PGE₂ works in part through the Wnt/β-catenin pathway, which is known to be important in promoting LGR5+ cell proliferation (63). PGE₂ prevents β-catenin degradation by inhibiting both the GSK-3β and Axin-2 functions thereby activating Wnt signaling (69). There are points of convergence of EGFR signaling and the Wnt/β-catenin pathway (65, 72). EGFR can activate β-catenin *via* the receptor tyrosine kinase-PI3K-Akt pathway (71).The regulation of β-catenin is downstream of Akt activation which is downstream of EGRF (73, 74). Although the evidence suggests that EGFR activation in response to TLR4 signaling is mediated by PGE₂, it is also possible that TLR4 signaling promotes EGFR activation through the production of amphiregulin, epiregulin or other EGFR ligands (75).

## Radioprotection

We have focused on nonmicrobial TLR activation as an early step in growth and wound repair but TLR activation also plays a role in radioprotection. In intestinal growth and wound repair the primary biologic event is LGR5+ stem cell proliferation; whereas, in radioprotection the primary biologic event is the prevention of radiation-induced apoptosis in LGR5+ stem cells. Both microbial and nonmicrobial TLR2 and TLR4 agonists induce radioprotection (76). As discussed earlier, orally administered LGG is radioprotective through release of the TLR2 agonist LTA (57). The synthetic TLR2 agonist PAM3-CSK4 is also radioprotective (57). LPS, a microbial agent, and HA, a nonmicrobial agent, are both radioprotective through a TLR4 mediated mechanism (44, 58). Each of these TLR2 and TLR4 agonists double the number of surviving small intestinal crypts after radiation suggesting that TLR2 and TLR4 activation are equally effective in promoting radioprotection.

The first step in the radioprotection induced by LGG, LTA or HA is TLR activation in pericryptal macrophages resulting in CXCL12 production and the migration of COX2-expressing MSCs from the lamina propria in the villi to a site near the epithelial cells in the base of the crypt (44, 56, 57). The first step in wound repair in DSS colitis is TLR4 activation in pericryptal macrophages resulting in the migration of COX-2 expressing MSCs from the lamina propria near the upper crypts to sites adjacent to epithelial cells in the lower crypts. Thus, the first step in radioprotection recapitulates the first step in wound repair. Administration of a radioprotective agent jump starts the wound repair process by inducing the first step in wound repair, the migration of PGE₂ producing MSCs to a site adjacent to LGR5+ stem cells. In radioprotection, PGE₂ blocks radiation-induced apoptosis in LGR5+ stem cells through transactivation of EGFR (48).

## Conclusions

The HA/macrophage TLR4/PGE₂/EGRF pathway mediates intestinal and colonic growth through LGR5+ stem cell proliferation and crypt fission (**Figure 1**). In this pathway, TLR4, which is usually associated with innate immunity, is activated not by the microbial product LPS, but by HA, a host molecule. Moreover, the TLR4 activation occurs not in the face of injury or “danger” but under homeostatic conditions. This pathway accounts for about 30% of intestinal and colonic growth (17, 40). It appears to be specific to the intestine and colon in that diminished elongation of the intestine and colon is the only observed growth defect in mice treated with PEP-1 from 3 to 8 weeks of age (40). It is not clear why this pathway is specific to the intestine and colon in that both HA and TLR4 are widely distributed. It is possible that the positioning of COX-2 expressing macrophages adjacent to epithelial stem cells is unique to the intestine and colon but this has not been addressed systematically.

In contrast to wound repair, where inflammation accompanies enhanced epithelial proliferation driven by TLR2/TLR4 activation (11, 12), in intestinal growth TLR4 activation promotes epithelial proliferation in the absence of inflammation (17). Moreover, in contrast to wound repair where activation of TLRs by both microbial PAMPs and non-microbial agents, such as HA, play a role (11, 12), intestinal growth is driven only by TLR4 activation by the nonmicrobial agent, HA (17).

The macrophage TLR4/CXCL12/MSC/PGE₂/EGFR pathway mediates colonic wound repair by promoting epithelial cell proliferation (**Figure 2**). In the repair phase of DSS colitis endogenous HA drives epithelial proliferation through TLR4 activation (22). Studies with antibiotics suggest that PAMPs released by commensal organisms also activate TLR2/TLR4 in DSS colitis (12). Irrespective of the agonist, macrophage TLR activation results in the migration of COX-2 expressing MSCs to sites near LGR5+ stem cells. Although MSCs are widely distributed, MSCs expressing COX-2 appear to be unique to the intestine and colon (77). FGF-10 released by intestinal and colonic epithelial cells induces COX-2 in MSCs (77). Intestinal MSCs express more than ten times as much COX-2 as macrophages. CXCL-12 produced by pericryptal macrophages mediates the migration of COX-2 expressing MSCs in LGG induced radioprotection (57). This is likely to also be the mechanism for the migration of COX-2 expressing MSCs in the repair phase of DSS colitis. LGR5+ stem cell proliferation is a key step in both growth and wound repair. In growth EGFR activation by PGE₂ accounts for about 30% of LGR5+ cell proliferation. The enhanced epithelial proliferation in the repair phase of DSS colitis is driven by PGE₂. Based on the growth studies, it is likely that EGFR activation by PGE₂ is also the mechanism of the increased epithelial proliferation in the repair phase of DSS colitis.

Wound repair mediated by HA activation of TLR2/TLR4 is also seen in the lung. TLR2/TLR4 activation by HA mediates wound repair in the bleomycin model of lung injury (29). This is a sterile inflammation demonstrating that inflammation driven by TLR activation can be part of the wound repair process even in the absence of microbial invasion. Whether PGE₂ is downstream from TLR activation in the bleomycin model has not been addressed; however PGE₂ is known to be therapeutic in this model (78). TLR4 activation by HA also affects the immune response in ischemia- reperfusion injury in the kidney and in acute allograft rejection in a skin transplant model (8).

TLR4 activation and inflammation promote wound repair. In sterile injury, as in bleomycin injury in the lung, TLR2/TLR4 activation is driven by non-microbial DAMPs, including LMW-HA (29). In non-sterile injury, as in DSS colitis, TLR2/TLR4 activation is driven both by PAMPs from commensal organisms and by LMW-HA released or exposed during injury (12, 22). In DSS colitis, TLR activation by PAMPs and TLR activation by HA are not mutually exclusive but rather are integrated components of wound healing. Although there are differences in the accessory molecules involved in TLR4 activation by LPS and LMW- HA, TLR4 activation by either one promotes wound healing (12, 27, 28, 33).

The macrophage TLR2-TLR4/CXCL12/MSC/PGE₂/EGFR pathway mediates radioprotection by exogenous TLR2/TLR4 agonists through inhibition of radiation induced apoptosis in LGR5+ stem cells (**Figure 2**). TLR2 agonists (LTA and PAM3-CSK4) and TLR4 agonists (LPS and HA) are radioprotective in the intestine (42, 57, 58). The radioprotective effects of LTA and HA depend on the migration of COX-2 expressing MSCs (57). PGE₂ mediates the final step in this radioprotection pathway, the inhibition of radiation-induced apoptosis in LGR5+ stem cells. PGE₂ binding to EP2 blocks radiation-induced apoptosis by an AKT-EGFR mechanism (48). Therefore it is likely that the intestinal radioprotection induced by TLR2 agonists and TLR4 agonists is mediated by EGFR activation, just as the enhanced LGR5+ stem cell proliferation in growth and wound repair goes through EGFR activation. PGE₂ is also radioprotective in the bone marrow (50). Several TLR agonists, including LPS, are radioprotective in the bone marrow (76). It is possible that bone marrow radioprotection induced by TLR agonist is also mediated by PGE₂ production but this question has not been addressed.

In mouse studies exogenous HA has effects on the GI tract that suggest potential therapies for human diseases. The pathways described here (**Figures 1** and **2**) provide multiple targets for pharmacologic interventions. Manipulation of these pathways by inhibitors of COX-2, CXCR4, and EGFR activation have been reviewed (17, 57). Exogenous HA induces intestinal hyperplasia through TLR4 activation (40). Thus, HA or other TLR4 agonists may be useful in short bowel syndrome. Exogenous HA acting through TLR2/TLR4 promotes wound repair in DSS colitis (22). HA, or other TLR2/TLR4 agonists, could promote wound repair in patients with GI injury from chemotherapy or radiation therapy. Exogenous HA has positive effects in infections with C. rodentium, raising the potential for use in GI infections in humans (43).

## Author Contributions

All authors contributed to the article and approved the submitted version.

## Funding

Crohn’s and Colitis Foundation Daniel H Present Senior Research Award, Ref. 370763, NIH grants (DK109384), philanthropic support from the Givin’ it all for Guts Foundation (https://givinitallforguts.org/), and The Lawrence C. Pakula MD IBD Innovation Fund.

## Conflict of Interest

The authors declare that the research was conducted in the absence of any commercial or financial relationships that could be construed as a potential conflict of interest.
